# On-board Training Strategy for IMU-Based Real-Time Locomotion Recognition of Transtibial Amputees With Robotic Prostheses

**DOI:** 10.3389/fnbot.2020.00047

**Published:** 2020-10-22

**Authors:** Dongfang Xu, Qining Wang

**Affiliations:** ^1^The Robotics Research Group, College of Engineering, Peking University, Beijing, China; ^2^Beijing Engineering Research Center of Intelligent Rehabilitation Engineering, Beijing, China; ^3^Beijing Innovation Center for Engineering Science and Advanced Technology, Peking University, Beijing, China

**Keywords:** robotic transtibial prosthesis, inertial measurement unit, on-board training, real-time recognition, human-machine interaction

## Abstract

The paper puts forward an on-board strategy for a training model and develops a real-time human locomotion mode recognition study based on a trained model utilizing two inertial measurement units (IMUs) of robotic transtibial prosthesis. Three transtibial amputees were recruited as subjects in this study to finish five locomotion modes (level ground walking, stair ascending, stair descending, ramp ascending, and ramp descending) with robotic prostheses. An interaction interface was designed to collect sensors' data and instruct to train model and recognition. In this study, the analysis of variance ratio (no more than 0.05) reflects the good repeatability of gait. The on-board training time for SVM (Support Vector Machines), QDA (Quadratic Discriminant Analysis), and LDA (Linear discriminant analysis) are 89, 25, and 10 s based on a 10,000 × 80 training data set, respectively. It costs about 13.4, 5.36, and 0.067 ms for SVM, QDA, and LDA for each recognition process. Taking the recognition accuracy of some previous studies and time consumption into consideration, we choose QDA for real-time recognition study. The real-time recognition accuracies are 97.19 ± 0.36% based on QDA, and we can achieve more than 95% recognition accuracy for each locomotion mode. The receiver operating characteristic also shows the good quality of QDA classifiers. This study provides a preliminary interaction design for human–machine prosthetics in future clinical application. This study just adopts two IMUs not multi-type sensors fusion to improve the integration and wearing convenience, and it maintains comparable recognition accuracy with multi-type sensors fusion at the same time.

## 1. Introduction

Robotic prosthetics plays an important role in assisting with the daily walking of lower-limb amputees. It can restore the functions of missed limb(s) and help to improve an amputee's balance and reduce the walking metabolic by adopting different control strategies (Au et al., 2009; Shultz et al., 2016; Feng and Wang, 2017; Kim and Collins, 2017). As the control strategies of robotic prosthetics depend on different locomotion modes or terrains, it is important to know the human locomotion mode accurately and efficiently. The question as to how we can acquire a locomotion mode has attracted a lot of attention over these years (Yuan et al., 2015; Zheng and Wang, 2016; Liu et al., 2017; Godiyal et al., 2018).

For lower-limb locomotion mode recognition, a surface electromyogram (sEMG) can record electrical potential generated by muscle cells and can be used to detect human locomotion intents, and this area has attracted plenty of attention and spurred much progress (Kim et al., 2013; Hargrove et al., 2015; Joshi et al., 2015; Afzal et al., 2016; Gupta and Agarwal, 2018). A mechanical sensor is also widely used in locomotion mode recognition. For example, the Inertial Measurement Unit (IMU) can provide position information (Ahmad et al., 2013; Young et al., 2014b; Bartlett and Goldfarb, 2018; Martinez-Hernandez and Dehghani-Sanij, 2018). Besides, a mechanical sensor is easily integrated with prosthetics. In addition, a capacitive sensing method has been applied in human locomotion mode recognition since it can measure muscle contraction and relaxation information directly (Zheng et al., 2014, 2017). Recently, some studies tend to fuse different sensor signals together to recognize locomotion intents and realize control (Novak and Riener, 2015) [e.g., sEMG signals and mechanical signals (Young et al., 2014a; Joshi and Hahn, 2016), mechanical signals and capacitive signals (Zheng and Wang, 2016), etc.].

Based on the sensing methods, studies about online locomotion mode recognition have been conducted (Elhoushi et al., 2017). Huang's research group has developed the locomotion modes recognition with four amputees wearing a hydraulic passive knee by fusing sEMG and mechanical signals, they and achieved 95% accuracy for recognizing seven tasks in real time (on Matlab) based on off-line model training (Zhang and Huang, 2013). Furthermore, they tried on-line recognition (on Matlab) based on sEMG and mechanical sensors with powered prosthetics (Zhang et al., 2015). Hargrove et al. have also developed on-line recognition by fusing sEMG and mechanical sensors (Spanias et al., 2016, 2018). We have also developed real-time on-board recognition of continuous locomotion modes for amputees with robotic transtibial prostheses and got more than 93% recognition accuracy with just two IMUs (Xu et al., 2018).

Though much progress has been made in on-line locomotion mode recognition, there still exist some problems to be solved. As is known, real-time recognition based on the off-line trained models is meaningful for the on-line control; however, off-line training brings a series of disadvantages for real-time recognition. Off-line training means bringing in other devices (e.g., a computer) to train the model, which is not convenient in integration with robotic prosthetics (Zhang et al., 2015; Xu et al., 2018). To improve the problem, Spanias et al. (2018) conducted a model updated on an embedded micro-controller based on mechanical sensor and sEMG. However, the multi-type sensors fusion method may bring wearing difficulty for amputees and integration difficulty for the prosthesis.

In this study, we put forward an on-board training model strategy for real-time recognition based on the robotic transtibial prosthesis using IMUs (IMUs are easily integrated with prosthesis) and develop a study of human locomotion mode recognition. This study is designed to recognize five locomotion modes [Level Ground walking (LG), Stair Ascending (SA), Stair Descending (SD), Ramp Ascending (RA), and Ramp Descending (RD)] based on the on-board trained model. A human–machine (prosthesis) interaction interface is designed to instruct to train model and recognize locomotion modes. The repeatability of gait signals, on-board training time, and recognition time are used to evaluate the performances based on different algorithms [Support Vector Machine (SVM), Quadratic Discriminant Analysis (QDA), and Linear Discriminant Analysis (LDA)]. Real-time recognition was conducted based on QDA.

## 2. Materials and Methods

### 2.1. Robotic Transtibial Prosthesis

**Figure 1:**
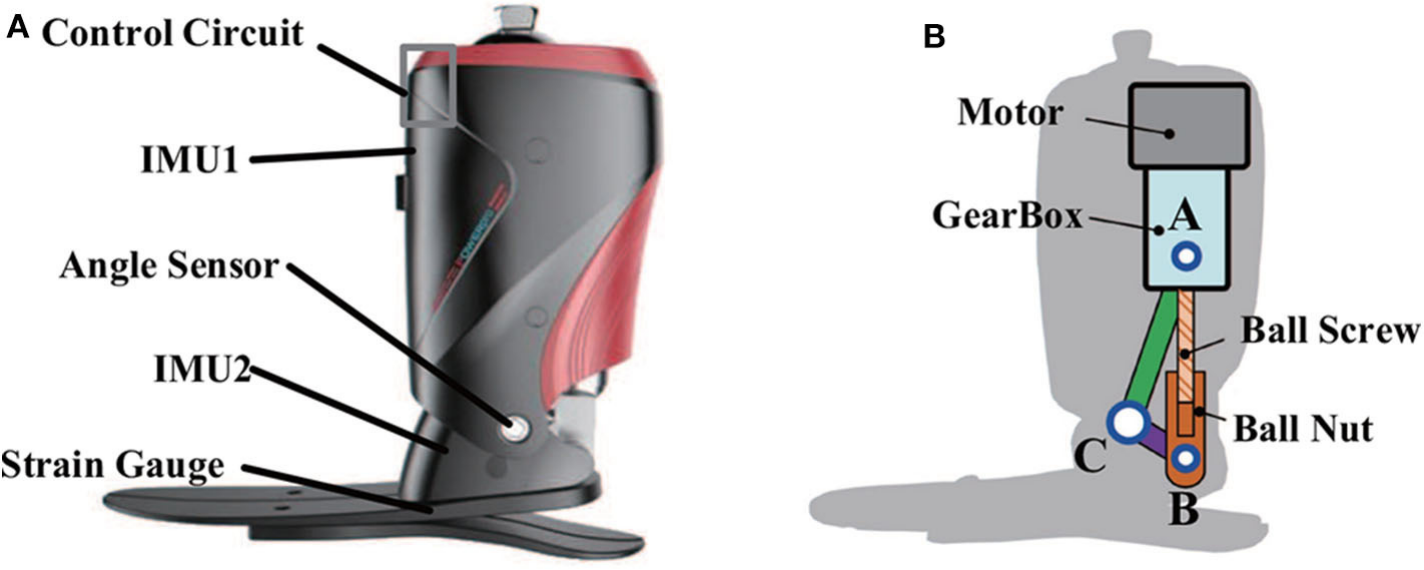
**(A)** The robotic transtibial prosthesis. **(B)** The three-bar ankle model: three bars (AB, BC, and AC) and three hinges (A, B, and C).

A commercialized version robotic prosthesis was used in this study (produced by SpeedSmart, a spin-off company of Peking University), as shown in Figure 1. The prosthesis model and other details can be seen in our previous studies (Wang et al., 2015; Feng and Wang, 2017). The prosthesis was comprised of one full bridge of strain gauge, one angle sensor, and two IMUs. One full bridge of strain gauge could reflect the deformation of the carbon-fiber foot, and the stance phase and swing phase could be detected based on the deformation information. Control strategies were performed based on the detected gait phases (Wang et al., 2015; Feng and Wang, 2017). One angle sensor was placed at the ankle of prosthesis to measure ankle's rotation with a 0–360° measurement range and a 12-bit resolution. Two IMUs were integrated on the prosthetic shank and foot. Each IMU included a triple-axis gyroscope (a measurement range of 0–2,000° with a resolution of 0.06°/s), a triple-axis accelerometer (a measurement range of 0–157 m/s^2^ with a resolution of 0.005 m/s^2^), and a triple-axis MEMS magnetometer (a measurement range of −4,800–4,800 μT with a resolution of 0.6 μT/LSB). IMU could provide the inclination angles (yaw, pitch, and roll), tri-axis acceleration, and tri-axis angular velocity information.

**Figure 2:**
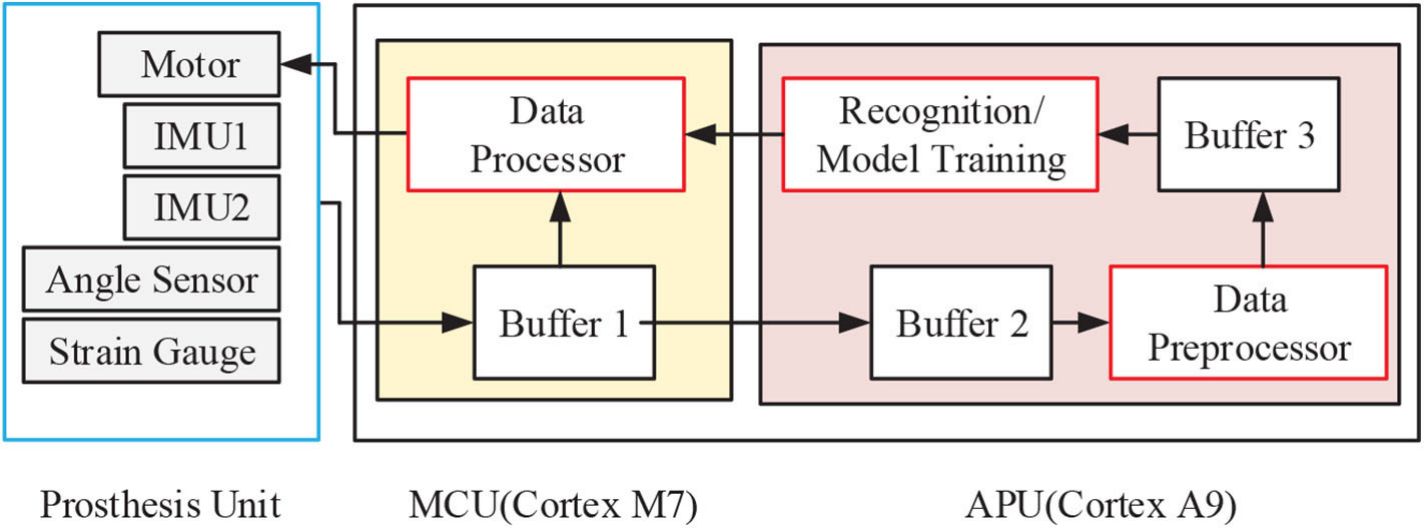
The control circuit of robotic transtibial prosthesis.

The control circuit of prosthesis consisted of a Micro Controller Unit (MCU) and an Application Processor Unit (APU), as shown in Figure 2. The MCU was based on a 216 MHz Cortex-M7 processer. The APU was constructed with an integrated programmable SoC chip, which consisted of two parts: a 667 MHz Cortex-A9 MPCore-based processing system (PS) and an FPGA-based programmable logic (PL) circuits. The MCU was used to collect and synchronize the prosthesis sensors signals, and it then packed them to the APU via Universal Asynchronous Receiver/Transmitter (UART). In addition, the MCU would send program instructions to the other prosthetic units. The APU was designed to execute on-board training and real-time recognition. APU would receive sensor data transmitted from the MCU by UART, and a micro SD card would be used to store data and trained model. The recognition results were packed together with sensor signals and then transmitted to a computer for further analysis in a wireless way.

### 2.2. Subjects

In this study, three transtibial amputees (Mean ± Standard Deviation, age: 45 ± 11.4 years, height: 170.3 ± 0.5 cm, and weight: 77 ± 5 kg) were recruited as subjects to finish the designed experimental tasks (S1, S2, and S3 represented the number of subjects). All subjects provided written informed consent forms. The experiments were approved by the Local Ethics Committee of Peking University.

### 2.3. Experimental Protocol

In the experiments, each subject lived with a prosthetic socket that would be mounted on the designed robotic prosthesis by adapter. To gain familiarity with and adapt to the new prosthesis and subsequent experimental tasks, all subjects would do some walking exercises before the experiments. The control parameters were adjusted according to the feedback of each subject ahead of the experiment.

**Figure 3:**
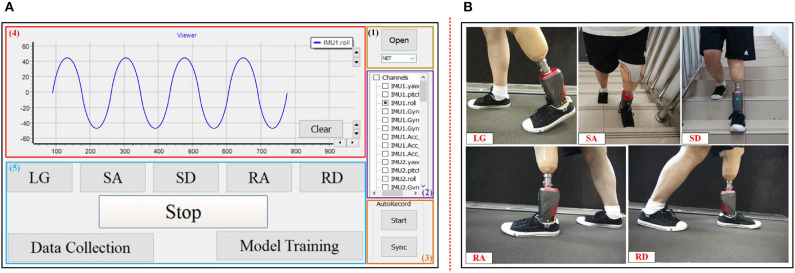
The designed interaction platform and experimental task of the study. **(A)** The designed interaction interface for the study. (1) Wireless connection option with prosthesis. (2) Signal channel. (3) Raw data recording. (4) Signal display. (5) Interactive operation for experimenter. **(B)** Subject wears the robotic prosthesis to finish five locomotion modes: LG, SA, SD, RA, and RD.

In this study, we first designed a preliminary interaction interface for the experiments as shown in Figure 3A. The interaction interface could send instructions to the prosthesis, and the prosthesis would return results to the interaction interface in a wireless way. The interaction interface included five parts. (1) The wireless connection with prosthesis to communicate with prosthesis. (2) Choosing signal channel(s) to plot signal curve(s). (3) Recording and saving raw data locally. (4) Signal display corresponding to the chosen channel in (2). (5) The interactive operation for experimenter. Figure 3B is a diagram of one amputee wearing a robotic prosthesis in the experiment.

The experiment could be divided into two sessions: (1) an on-board training session and (2) a real-time recognition session. The experimental duration was about 2 h for each subject. In the on-board training session, each subject was instructed to accomplish the five modes, including the LG, RA, RD, SA, and SD in sequence. All subjects were asked to walk on the treadmill at their self-selected speeds for collecting data on the LG mode. For the RA and RD, the subjects would walk on the treadmill at an incline of 10° at their self-selected speeds. The subjects accomplished SA and SD on the stairs with a height of 16 cm and a length of 28 cm at their normal walking speeds. For each locomotion mode, 20 s of data were collected in the training session for training model. The real-time recognition experiment in session (2) was conducted based on the trained model in session (1).

In the experiment, we could perform data collection for training models with corresponding modes (i.e., label) by use of the interaction operation. When the experiment started, the subject would walk in a steady locomotion mode (for example LG), and we clicked the corresponding mode (corresponding to “LG”) and then clicked “Data Collection” in (5) of Figure 3A to collect sensor signals with labels as training data. After finishing data collection, we clicked the “Stop” option and finished training data collection. Then “Model Training” was clicked to send instructions to the on-board system (i.e., control circuit) of the prosthesis to start on-board training of the model. The real-time recognition would start as soon as the on-board training was finished. During real-time recognition, the recognition tasks were the same as the training tasks, namely, finishing the five locomotion modes: LG, SA, SD, RA, and RD.

### 2.4. Signal Processing

**Figure 4:**
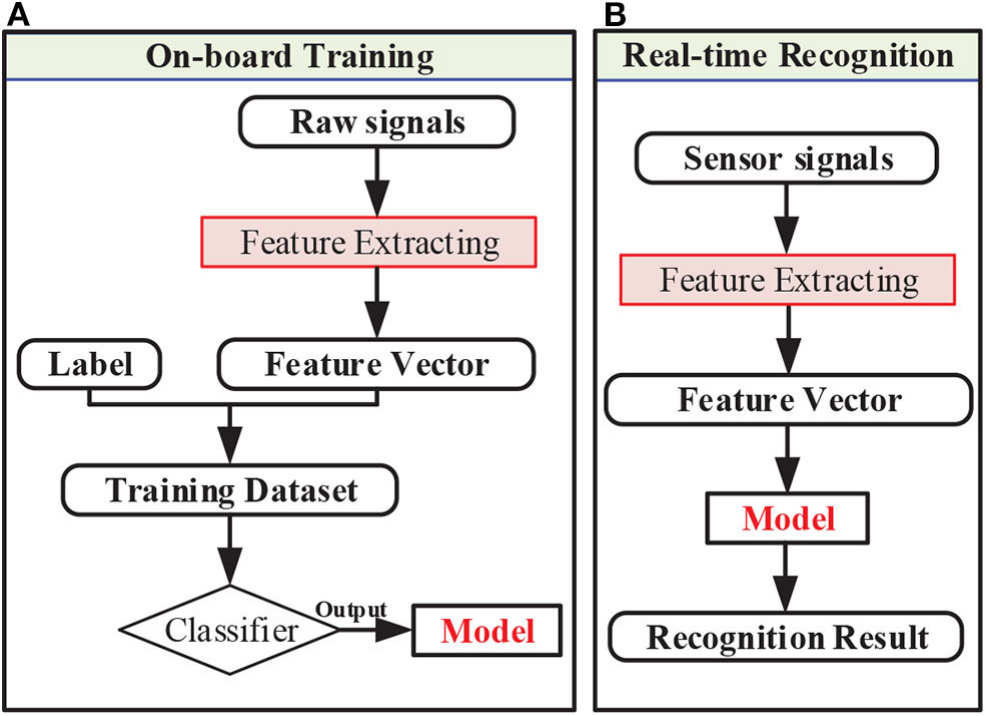
The procedures of signal processing for **(A)** on-board training and **(B)** real-time recognition.

Signal processing is important to the on-board training and recognition. The main procedures of on-board training and recognition could be seen in Figure 4. For both training and recognition, raw signals needed to be processed to extract features. Feature extracting was performed based on raw signal data by sliding window. For on-board training, feature vectors and labels formed a training data set and were then used for the training mode. After on-board training was finished, the continuous feature vectors were fed into the model for recognition one by one in time sequence. The feature extracting method and recognition algorithm could been seen as follows.

#### 2.4.1. Signal Feature Extracting

Two IMUs were integrated in the robotic prosthetic shank and foot. IMU could provide the raw signals of nine channels as mentioned: inclination angles (yaw, pitch, and roll), tri-axis acceleration, and tri-axis angular velocity signals. The sample frequency was 100 Hz. The pitch and roll angle of IMU can be calculated according accelerometer, and the yaw angle can be calculated according the MEMS magnetometer and accelerometer, but it needs static calibration to remove the drift. We chose IMU information from eight channels (angles from two channels, triple-axis acceleration, and triple-axis angular velocity), excluding yaw angle information. A sliding window was selected to extract features of raw signals. Each window's length was 250 ms and the sliding increment was 10 ms (Zheng and Wang, 2016; Xu et al., 2018). Five time domain features were selected for this study. These features were *f*1 = *mean*(*Y*), *f*2 = *std*(*Y*), *f*3 = *max*(*Y*), *f*4 = *min*(*Y*), and *f*5 = *sum*(*abs*(*diff*(*Y*))), where Y was the data matrix of one sliding window, and the data sizes of one sliding window were 25 (250 ms' length) by 16 (16 channels in total). The *mean*(*Y*) and *std*(*Y*) were the average value and the standard deviation of each channel in *Y*, respectively. The *max*(*Y*) and *min*(*Y*) were the maximum and minimum of each channel in *Y*, respectively. The *diff*(*Y*) was the difference value of adjacent two elements of each channel in *Y*. The *sum*(*Y*) was the summation of each channel in *Y*. The *abs*(*Y*) was the absolute value. All these feature values were concatenated together to be a feature vector (we did not conduct a feature vector dimension reduction). Feature vectors with labels constituted a training data set, and the model was trained based on the training data set. For recognition, the raw IMU signals were also processed following the same processing procedures and one feature vector was generated each time. The continuous feature vectors were fed into the trained model and then we could get the recognition results.

#### 2.4.2. Recognition Algorithm

Recognition algorithms directly affect the recognition accuracy. SVM, QDA, and LDA were widely used in locomotion mode recognition (Liu et al., 2017). In the previous study, we have compared the on-board recognition performances of different algorithms (SVM, QDA, and LDA) (Xu et al., 2018). SVM algorithm could achieve high recognition accuracy, LDA could achieve good recognition time performance, and QDA could take count of accuracy and recognition time performance (Xu et al., 2018). For multi-class (five locomotion modes in this study) recognition, a one vs. one strategy was adopted. Here we conducted this study based on the three algorithms.

The core of SVM is to construct an optimal hyperplane to separate the data belonging to different classes. The hyperplane equation is as follows:

(1) $$\begin{array}{r} w\cdot x+b=0 \end{array}$$

where *w* is the weight vector, *b* is the constant item (bias), and *x* is the input vector (i.e., feature vector). To construct this hyperplane, we need to optimize the objective function, and the objective function is as follows:

(2) $$\begin{array}{r} \begin{array}{c} \underset{w,x}{\min}\left(\frac{1}{2}||w|{|}^{2}+C\left(\overset{N}{\underset{i=1}{\sum }}\xi_{i}\right)\right)\\ s.t.\;y_{i}\left[w*x_{i}+b\right]\geq 1-\xi_{i}\\ \xi_{i}\geq 0,i=1,2,...,N \end{array} \end{array}$$

where *C* is the penalty parameter (the default of *C* is 1) that represents penalty for misclassification, and its function is adjusting the confidence interval range. *N* is the number of training data samples, ξ_*i*_ is the relaxation factor corresponding to the *i*_*th*_ training data sample (*x*_*i*_). *y*_*i*_ (*y*_*i*_ = 1 or −1) is the label corresponding to *x*_*i*_. Its discriminant function can be seen as follows (Vapnik and Hervonenkis, 1974):

(3) $$\begin{array}{r} f\left(x\right)=sgn\left(\overset{N}{\underset{i=1}{\sum }}\alpha_{i}y_{i}K\left(x_{i},x\right)+b\right) \end{array}$$

where α_*i*_, *x*_*i*_, and *b* mainly construct the SVM model for the study, which need to be trained on board, and they denote coefficient, support vector, and constant terms, respectively. *K*(*x*_*i*_, *x*) is the kernel function of SVM. In our study, a radial basis function is chosen as the kernel function of SVM to realize on-board training and recognition. It is shown as follows:

(4) $$\begin{array}{r} K\left(x_{i},x\right)=e^{\gamma ||x_{i}-x|{|}^{2}} \end{array}$$

where γ is a coefficient that determines the distribution of data mapped to a new feature space, and its function is adjusting the effect of each sample on the classification hyperplane [γ = 1/*n* (default), where *n* is the feature vector's dimension, *n* = 80].

QDA and LDA are based on normal distribution hypothesis (Friedman, 1989). It is assumed that the feature vectors are multivariate normally distributed with estimated specific mean vector μ, and covariance matrix Σ of each class data for QDA. In this study, the number of class (locomotion modes) is five. For the QDA algorithm, its discriminant function is

(5) $$\begin{array}{r} f\left(x\right)=x\cdot W\cdot x^{T}+w\cdot x^{T}+w_{0} \end{array}$$

Here, *W*, *w*, and *w*_0_ construct the QDA model, and they are the functions of the estimated specific mean vector and covariance matrix of each class, which could be denoted as

(6) $$\begin{array}{r} W=-\frac{1}{2}\cdot \left(\Sigma_{i}^{-1}-\Sigma_{j}^{-1}\right) \end{array}$$

(7) $$\begin{array}{r} w=\mu_{i}\cdot \Sigma_{i}^{-1}-\mu_{j}\cdot \Sigma_{j}^{-1} \end{array}$$

(8) $$\begin{array}{r} w_{0}=-\frac{1}{2}\cdot \left(\mu_{i}\cdot \Sigma_{i}^{-1}\cdot \mu_{i}^{T}-\mu_{j}\cdot \Sigma_{j}^{-1}\cdot \mu_{j}^{T}\right)-\frac{1}{2}\cdot ln\frac{\left|\Sigma_{i}\right|}{\left|\Sigma_{j}\right|} \end{array}$$

Here, Σ_*i*_, μ_*i*_, Σ_*j*_, and μ_*j*_ are the estimated specific mean vector and covariance matrix corresponding to class *i* and class *j*, respectively. |Σ_*i*_| and |Σ_*j*_| are the determinants of Σ_*i*_ and Σ_*j*_. In the study, all these parameters are estimated on board to construct QDA model.

For LDA, it is assumed that the feature vectors are multivariate, normally distributed with an estimated mean vector of each class data and common covariance matrices (Σ_1_ = Σ_2_ = …= Σ) for LDA (Friedman, 1989). Σ can be denoted as

(9) $$\begin{array}{r} \Sigma =\overset{C}{\underset{i=1}{\sum }}\frac{N_{i}-1}{N-1}\Sigma_{i} \end{array}$$

where *C* is the number of classes, *N*_*i*_ is the number of feature vectors of class *i*, and *N* is the number of total feature vectors (*N* = *N*_1_ + *N*_2_ + … + *N*_*i*_ + *N*_*C*_). The discriminant function of LDA can be denoted as

(10) $$\begin{array}{r} f\left(x\right)=w\cdot x^{T}+w_{0} \end{array}$$

Here, *w* and *w*_0_ could be denoted as

(11) $$\begin{array}{r} w=\left(\mu_{i}-\mu_{j}\right)\cdot \Sigma^{-1} \end{array}$$

(12) $$\begin{array}{r} w_{0}=-\frac{1}{2}\cdot \left(\mu_{i}+\mu_{j}\right)\cdot \Sigma^{-1}\cdot {\left(\mu_{i}-\mu_{j}\right)}^{T} \end{array}$$

The LDA model is constructed of *w* and *w*_0_ without *W* compared with QDA and it is a linear function of *x*, which is trained on board with a lower computation amount than QDA model in the study.

### 2.5. System Evaluation

In this study, the repeatability of gait signals was analyzed. The time performance, recognition accuracy, and classifier quality were also evaluated.

#### 2.5.1. The Repeatability of Signals

The movement of the lower limb is periodical or quasi-periodical. The repeatability of signals can reflect the repeatability of gait waveforms over gait cycles (Godiyal et al., 2018). The variance ratio (VR) is a widely used metric to analyze the repeatability of signals (Erni and Colombo, 1998; Hwang et al., 2003; Godiyal et al., 2018). VR is expressed as follows (Hershler and Milner, 1978):

(13) $$\begin{array}{r} VR=\frac{\frac{1}{n}\sum_{i=1}^{n}\left(\frac{1}{N-1}\sum_{j=1}^{N}{\left(X_{ij}-{\bar{X}}_{i}\right)}^{2}\right)}{\frac{1}{n-1}\sum_{i=1}^{n}\sum_{j=1}^{N}{\left(X_{ij}-\bar{X}\right)}^{2}} \end{array}$$

where *N* denotes the number of gait cycles. For each gait cycle, signals is normalized by interpolation and has a fixed length, namely, *n* (*n* is 1,000 in the study). *X*_*ij*_ is the *i*_*th*_ shank angle signal value in *j*_*th*_ gait cycle. ${\bar{X}}_{i}$ is the mean of signals at *i*_*th*_ data point over *N* gait cycles, and $\bar{X}$ is the mean of ${\bar{X}}_{i}$ over the gait cycle. ${\bar{X}}_{i}$ and $\bar{X}$ are formulated as

(14) $$\begin{array}{r} {\bar{X}}_{i}=\frac{1}{N}\overset{N}{\underset{j=1}{\sum }}X_{ij} \end{array}$$

(15) $$\begin{array}{r} \bar{X}=\frac{1}{n}\overset{n}{\underset{i=1}{\sum }}{\bar{X}}_{i} \end{array}$$

VR can measure the degree of dispersion of data, and it varies from 0 to 1. When VR is close to 0 in the study, it means high repeatability of the IMU signals, which reflects the repeatability of gait.

#### 2.5.2. Time Performance Evaluation

The training process started as the experimenter pressed the “Model training” button (as shown in Figure 3A) and ended with model saving. The training time consisted of model generating and saving time. After the training process ended and the model was generated and saved, the real-time recognition started to run based on the trained model. Recognition was continuous and multiple based on various feature vectors streams. For each recognition, the recognition process was that one feature vector was fed into the trained model, and the recognition result was then outputted. The recognition decision consist of collections of the data, preparation of the feature vector, and the recognition process. The time to execute the recognition decision was recorded as recognition time.

#### 2.5.3. Recognition Accuracy Evaluation

Recognition accuracy was an important metric to evaluate the recognition performance. The recognition accuracy for each locomotion mode could be denoted as follows:

(16) $$\begin{array}{r} c_{ij}=\frac{n_{ij}}{n_{i}}\times 100\% \end{array}$$

where *n*_*ij*_ was the number that test samples (belonging to mode *i*) were recognized as mode *j*, and *n*_*i*_ was the number of the total test samples belonging to mode *i*. The confusion matrix (CM) was also used to evaluate the recognition performance for each mode in detail, which is shown as follows:

(17) $$\begin{array}{r} CM=\left(\begin{array}{cccc} c_{11} & c_{12} & ... & c_{1m}\\ c_{21} & c_{22} & ... & c_{2m}\\ ... & ... & c_{ij} & ...\\ c_{m1} & c_{m2} & ... & c_{mm} \end{array}\right) \end{array}$$

The *m* denotes the number of locomotion modes. The element*c*_*ij*_ in confusion matrix *CM* is shown in Equation (16). The diagonal elements in *CM* denoted the recognition accuracy of each mode.

#### 2.5.4. Receiver Operating Characteristic

The receiver operating characteristic (ROC) could check the quality of classifiers and was used to evaluate the locomotion mode recognition in this study. For each class of a classifier, the ROC applies threshold values across the interval [0,1] to outputs. For each threshold, two values [the True Positive Ratio (TPR) and the False Positive Ratio (FPR)] were calculated. TPR is the predict/recognition accuracy for class *i* (i.e., locomotion mode *i*), and FPR is the number of samples whose actual class is not class *i*, but predicted to be class *i*, divided by the number of outputs whose predicted class is not class *i*. Then we can get a series of TPR and FPR pairs, which forms the ROC curve. The more each curve hugs the left and top edges of the plot, i.e., the bigger the area under the ROC curve (AUC) the better the classification.

## 3. Results

### 3.1. Repeatability of Gait Signals

**Figure 5:**
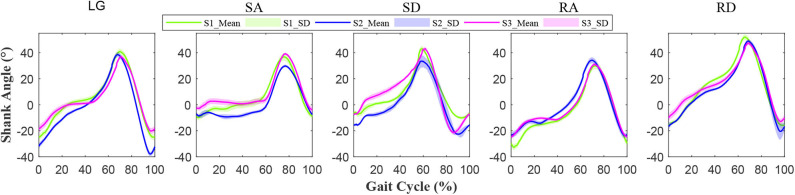
Normalized angle signals of the prosthetic shank. The horizontal axis represents the gait cycle. One gait cycle is from heel strike to toe off and ends at the next heel strike of the prosthesis. The vertical axis denotes the shank angle of prosthesis relative to the vertical direction. The solid line shows the mean and the shaded area represents the standard deviation (SD) of the signal. The different colors represent different subjects (S1, S2, and S3), as shown in the legend.

The normalized shank angle of prosthesis relative to the perpendicular to the ground was shown in Figure 5. The solid line shows the mean, and the shaded area represents the standard deviation (SD) of the signal. The mean and standard deviation showed the quasi-periodicity of lower-limb movement. From Figure 5, it could be seen that each subject had their specific signal feature.

The repeatability of gait signals (waveforms) over gait cycles was analyzed based on variance ratio, as listed in Table 1. For LG and SA, the shaded area corresponding to S2 (in Figure 5) was smaller on the whole, and S2 could achieve smaller variance ratio values (0.006 and 0.01), as shown in Table 1, which indicated better repeatability than S1 and S3. For SD, RA, and RD, S2 could achieve bigger variance ratio values (0.051, 0.015, and 0.021), and its shaded area (in Figure 5) was bigger; S1 and S3 thus achieved better repeatability than S2.

**Table 1:** The repeatability of gait signals (waveforms) of subjects based on variance ratio analysis.

	**LG**	**SA**	**SD**	**RA**	**RD**
S1	0.013	0.029	0.011	0.013	0.008
S2	0.006	0.010	0.051	0.015	0.021
S3	0.014	0.023	0.008	0.006	0.014

### 3.2. On-board Training and Recognition Time

**Figure 6:**
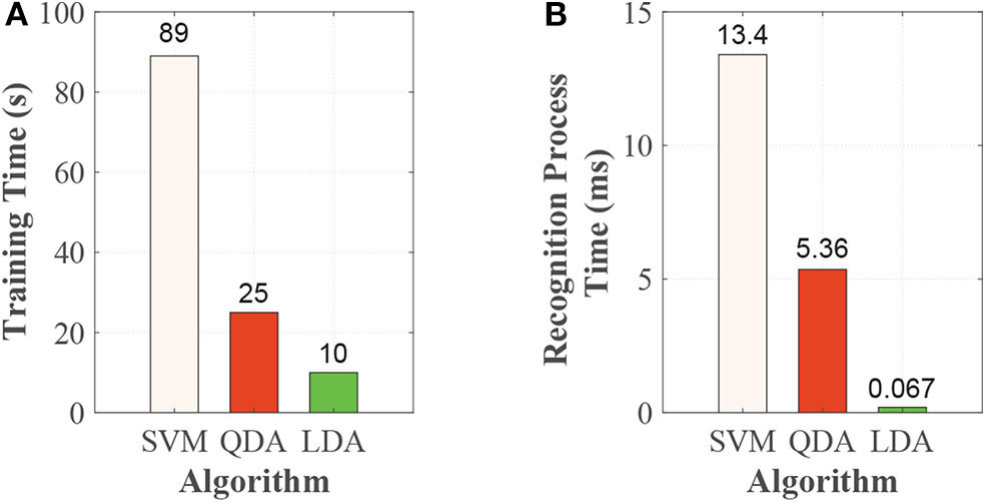
The time performances based on SVM, QDA, and LDA. (**A**) Training time and (**B**) Recognition process time.

The APU of prosthesis control circuit was designed to execute on-board training and real-time recognition. The on-board training and recognition times based on the APU are shown in Figure 6. On-board training time was related to the size of training data set. The acquisition of training data set was as follow. For each sampling, we could get one feature vector and each vector contained 80 values (two IMUs, eight channels of each IMU, and five feature values of each channel's signal, 2 × 8 × 5 = 80). In this study, we asked each subject to finish the five locomotion modes (LG, SA, SD, RA, and RD), and collected 2,000 feature vectors for each locomotion modes. After this, we could get 10,000 feature vectors corresponding five locomotion modes. The training data set consisted of a matrix and its size was 10,000 × 80. The training time consisted of model generating and saving time, which were 89, 25, and 10 s for SVM, QDA, and LDA, respectively, as shown in Figure 6A.

The recognition decision was comprised of data collection, feature vector preparation, and the recognition process. It took <1 μs for data collection and 0.146 ms for preparation of feature vector each time. The data collection and preparation times of the feature vector were the same for SVM, QDA, and LDA. For recognition, when the each subject walked, we could get one feature vector for each sampling, and each feature vector contained 80 values. By feeding the feature vector streams into the trained model, we could get the continuous recognition results. The recognition process was from one feature vector fed into the model to output the result. The recognition process time were 13.4, 5.36, and 0.076 ms, corresponding to SVM, QDA, and LDA, respectively, as shown in Figure 6B.

### 3.3. Recognition Accuracy

The real-time recognition was conducted based on QDA and the off-line recognition was conducted based on LDA and SVM. Real-time recognition and off-line recognition are conducted using the same training data and test data. The total real-time recognition accuracies for the three subjects were 96.51, 97.33, and 97.73%, and the mean accuracy and SEM (standard error of mistake) was 97.19 ± 0.36% based on QDA, as shown in Table 2. The off-line recognition accuracies and SEMs (standard error of mistake) were 89.96 ± 0.48%, and 97.11 ± 0.93% based on LDA and SVM, as shown in Table 2, respectively.

**Table 2:** Recognition accuracy (mean ± SEM) based on QDA, LDA, and SVM.

**Subjects**	**QDA**	**LDA**	**SVM**
	**(real-time) (%)**	**(off-line) (%)**	**(off-line) (%)**
S1	96.51	90.50	97.05
S2	97.33	89.01	98.74
S3	97.73	90.37	95.53
Mean ± SEM	97.19 ± 0.36	89.96 ± 0.48	97.11 ± 0.93

**Figure 7:**
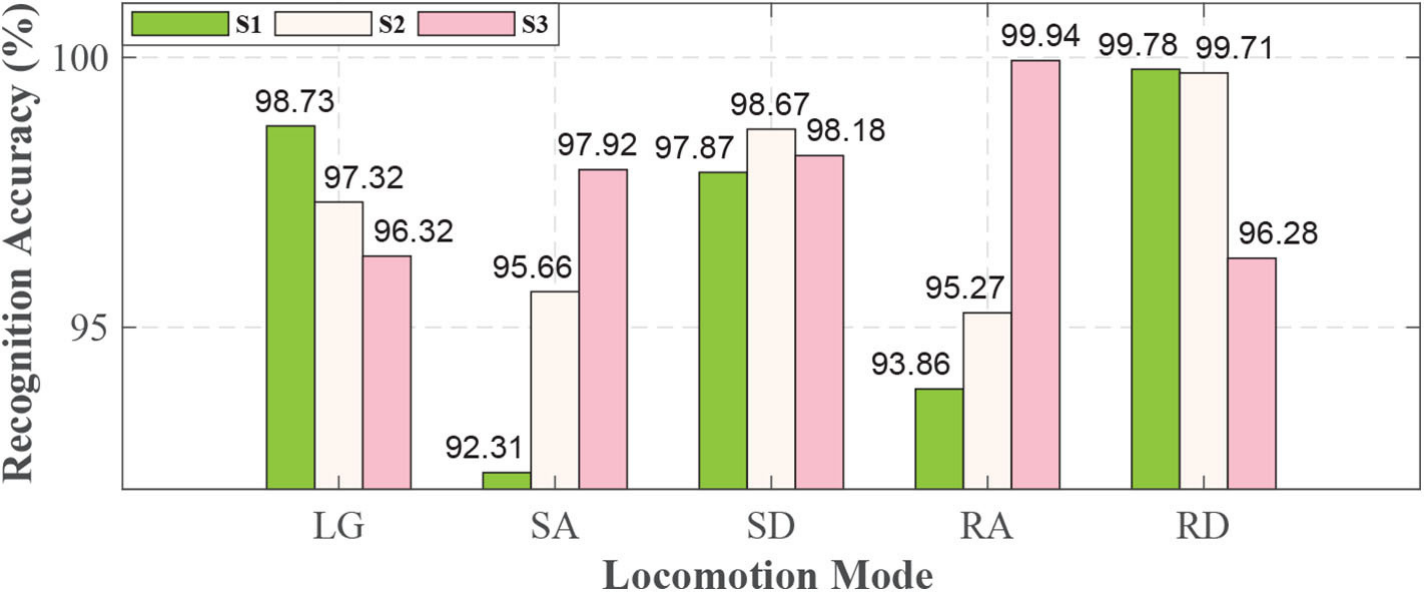
Real-time recognition result for each locomotion mode based on QDA algorithm. The height of the bar denotes the recognition accuracy. The different colors of bars denote subjects as the legend shows.

For each locomotion mode, it could be recognized with more than 92% accuracy for each subject, as shown in Figure 7.

For S1 and S2, this study could achieve the highest recognition accuracies (99.78 and 99.71%, respectively) in RD and lowest recognition accuracies in SA (92.31%) and RA (95.27%). For S3, it could achieve the highest recognition accuracy in RA (99.94%) and the lowest recognition accuracy in RD (96.28%). There were some differences among the subjects for each locomotion mode.

A confusion matrix (mean ± SEM) was used to evaluate the recognition performance for each locomotion mode, as listed in Table 3. In Table 3, we could see that each locomotion mode could be recognized with quite high accuracy, and the SEM is no more than 2%. The highest accuracy was achieved in recognizing RD (98.59 ± 1.16%), and the lowest was achieved in recognizing SA (95.30 ± 1.63%). From Table 3, we could see that LG was mistakenly recognized as RD (1.87 ± 0.57%), SA was mistakenly recognized as SD (3.64 ± 1.61%), SD was mistakenly recognized as RD (1.05 ± 0.54%), RA was mistakenly recognized as LG (2.64 ± 1.82%), and RD was mistakenly recognized as SD (1.34 ± 1.19%).

**Table 3:** Recognition confusion matrix (mean ± SEM) based on QDA (%).

	**LG**	**SA**	**SD**	**RA**	**RD**
LG	97.46 ± 0.07	0.00 ± 0.00	0.23 ± 0.23	0.45 ± 0.28	1.87 ± 0.57
SA	0.15 ± 0.15	95.30 ± 1.63	3.64 ± 1.61	0.02 ± 0.02	0.89 ± 0.70
SD	0.68 ± 0.68	0.03 ± 0.03	98.24 ± 0.23	0.00 ± 0.00	1.05 ± 0.54
RA	2.64 ± 1.82	0.00 ± 0.00	0.48 ± 0.45	96.36 ± 1.84	0.52 ± 0.52
RD	0.07 ± 0.04	0.00 ± 0.00	1.34 ± 1.19	0.00 ± 0.00	98.59 ± 1.16

### 3.4. Receiver Operating Characteristic

**Figure 8:**
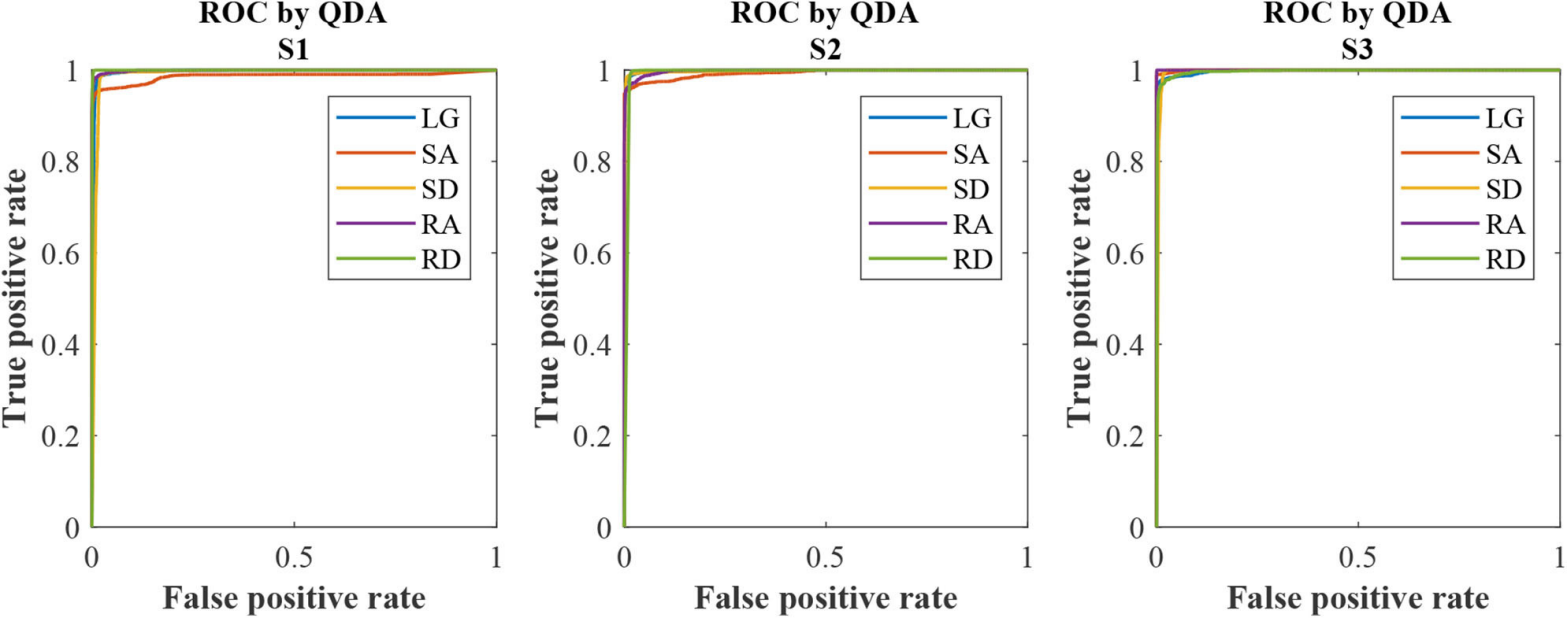
ROC curves for the subjects (S1, S2, and S3) based on QDA. The color of solid line denotes locomotion mode as the legend shows.

The ROC curves can be seen in Figure 8. We could see that each curve hugs the left and top edges of the plot. The AUC for each subject and for the five locomotion modes (LG, SA, SD, RA, and RD) can be seen in Table 4. For S1, the AUCs for SA were 0.9865 and 0.9957, 0.9915, 0.9991, and 0.9996, for LG, SD, RA, and RD, respectively. For S2 and S2, the AUCs were more than 0.99 for each locomotion mode, as shown in Table 4. The maximum AUC was 0.9865 (S1, LG) and the maximum AUC was 1.0 (S3, RA).

**Table 4:** The AUC for each locomotion mode and each subject based on QDA.

**Subjects**	**LG**	**SA**	**SD**	**RA**	**RD**
S1	0.9957	0.9865	0.9915	0.9991	0.9996
S2	0.9985	0.9933	0.9974	0.9980	0.9935
S3	0.9982	0.9996	0.9961	1.0	0.9969

### 3.5. Robustness of Recognition

**Figure 9:**
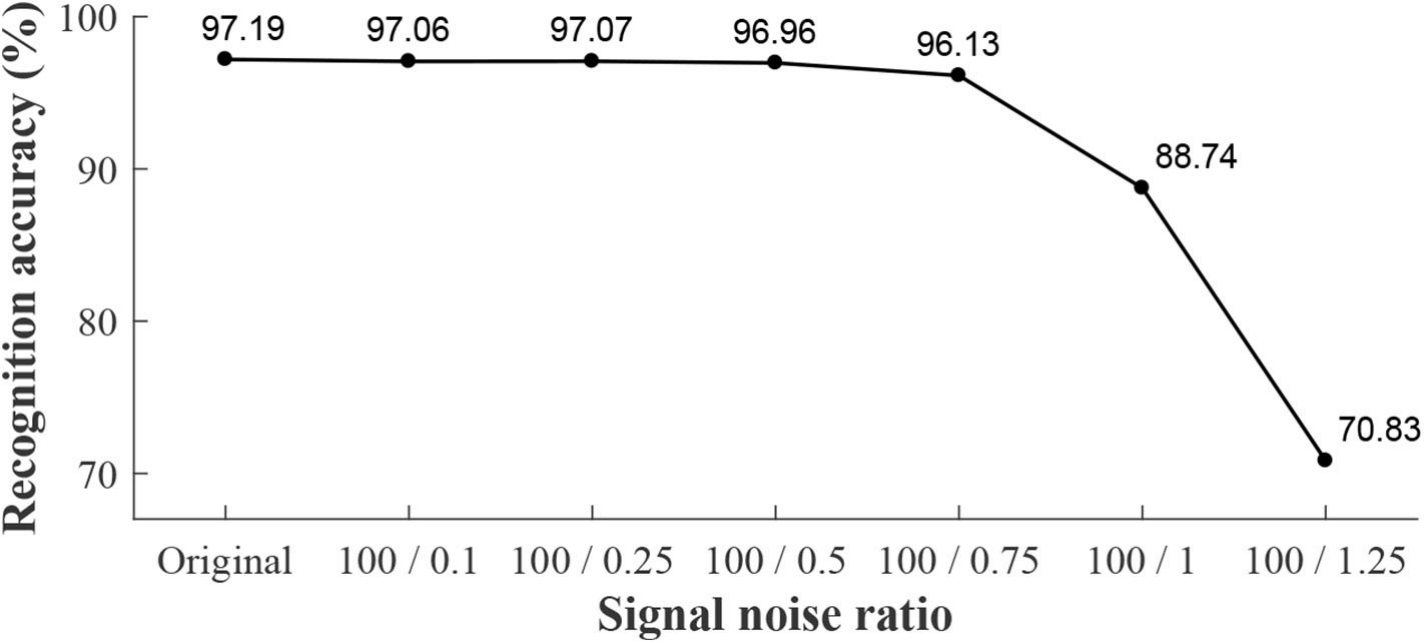
Robustness analysis (off-line) of recognition for locomotion modes. The horizontal axis denotes the signal noise ratio, which represents the noise added by manual into original data. Here, “Original” denotes the original data without added noise. The text in this figure denotes the recognition accuracy.

We conducted a robustness analysis of recognition, which was done by adding some white noise artificially to the data and running the experiments in off-line mode. The signal noise ratios were 100 : 0.1, 100 : 0.25, 100 : 0.5, 100 : 0.75, 100 : 1, and 100 : 1.25. To make comparisons, we conducted recognition analysis in the off-line mode, and the off-line results combined with real-time recognition accuracy can be seen in Figure 9. Real-time recognition based original signals without added noise was 97.18% (as mentioned above). The off-line recognition results with added noise were 97.06, 97.07, 96.96, 96.13, 88.74, and 70.83%, corresponding to different signal noise ratios (100 : 0.1, 100 : 0.25, 100 : 0.5, 100 : 0.75, 100 : 1, and 100 : 1.25, respectively). When the signal noise ratio was no <100 : 0.75, the recognition accuracy was more than 96.0%.

## 4. Discussion

Most of the current locomotion modes recognition is based on off-line trained model. Off-line training will bring other devices (e.g., a computer) to the training model, which is not convenient to integrate with robotic prosthesis. In addition to the off-line problem, some multi-type sensors fusion method for improving locomotion mode accuracy may bring wearing and integration difficulties. In this paper, on-board training and real-time locomotion modes recognition has been conducted using two IMUs of robotic transtibial prostheses. We have design an interactive interface to execute the on-board training and recognition.

### 4.1. The Repeatability of Signals

The repeatability of signals can reflect gait features, and it is affected by a lot of factors, such as sensor types, movement regularity of different subjects, and so on (Hwang et al., 2003; Godiyal et al., 2018). Compared with force myography and sEMG signals (Hwang et al., 2003; Godiyal et al., 2018), the shank angle (IMU signals) of prosthesis relative to perpendicular to the ground are used to evaluate the repeatability of lower-limb movement. Figure 5 and Table 1 show how all the locomotion modes show good repeatability (VR is no more than 0.051), which is comparable to (and even better than) the other research (Godiyal et al., 2018). The repeatability of signal waveforms is important for training and recognition. Good repeatability of signals may reduce the amount of training data and provide supports for locomotion recognition or gait prediction.

### 4.2. Training and Recognition Time for Different Algorithms

The on-board training time and recognition decision time are affected by the performances of hardware, algorithm, and so on. In this study, we conducted the experiment to analyze the performances of different algorithms (SVM, QDA, and LDA) based the same on-board hardware system and experimental training and test data. The time performance can reflect the computation efficiency of system and the complexity of algorithms. The training time in our study are 89 s for SVM, 25 s for QDA, and 10 s for LDA. For SVM, model training requires continuous iterative optimization, which is very time consuming. Training with SVM requires more time than QDA and LDA due to its complexity. For QDA, the mean vector and covariance matrix of training data are evaluated, and the inverse matrix and determinant of covariance matrix are computed in the training process. Training with LDA need less time for its lower algorithm complexity compare with QDA, as shown in Equations (11) and (12). The recognition decision process was comprised of collection of the data, preparation of the feature vector and the recognition process. It took <1 μs for data collection and 0.146 ms for preparation of the feature vector each time. Data collection time and preparation time of feature vector were same for SVM, QDA, and LDA. The recognition process time are 13.4, 5.36, and 0.067 ms, corresponding to SVM, QDA, and LDA. For locomotion mode recognition, the recognition process time should be less than sliding window increment (Englehart and Hudgins, 2003), which can void data collision and leave time for subsequent process. In our experiments, the sliding window increment is 10 ms, which means the recognition decision must be finished within 10 ms, and QDA and LDA are thus better than SVM. Our previous study has used QDA taking count of accuracy and recognition time performance (Xu et al., 2018). In this study, we also use QDA in recognition study.

### 4.3. Recognition Accuracy Based on QDA

The real-time recognition accuracy (based on QDA) and the off-line recognition accuracy (based on LDA and SVM) are conducted. The results show that we can get high accuracy (more than 97%) when using QDA and SVM. While using LDA, the accuracy is no more than 90%. The mean real-time recognition accuracies for each locomotion mode are more than 95% and the standard error of mistakes are no more than 2%, as seen in Table 3, which is comparable with current online recognition studies (Zhang et al., 2015; Spanias et al., 2018; Xu et al., 2018). Besides, the high accuracy and low standard error of mistake show the feasibility of this study.

From the Table 3, LG is mistakenly recognized as RD with 1.87% error, and RA is mistakenly recognized as LG with 2.64% error, which shows some confusion trends among the three locomotion modes (LG, RA, and RD). As is known, level ground and ramps are even terrains, and the similarities between the terrains may cause confusion when it comes to recognizing the three locomotion modes (LG, RA, and RD) (Spanias et al., 2018). We also note that SA is mistakenly recognized as SD with 3.64% error because of the similarity between the terrains (stairs). In Table 3, it also shows SD is recognized as RD with 1.05% error, and RD is recognized as SD with 1.34% error. As we know, the subject is in downward ambulation direction no matter he is in SD or RD, so there are some recognition errors between SD and RD. Spanias et al. (2018) conducted model updating on an embedded micro-controller based on a mechanical sensor and sEMG, and they view LG and SA as one class. Compare with the study, we made progress in terms of integration and recognition accuracy. In this study, just two IMUs are adopted, because IMU is more easily integrated with prosthesis. Besides, our study can discriminate LG and SA and also achieve comparable effect with some other studies (Zhang et al., 2015; Xu et al., 2018).

We have used ROC and AUC to check the quality of QDA classification algorithm. Each ROC curve (in Figure 8) hugs the left and top edges of the plot and the AUCs (in Table 4) for each subject and each locomotion mode are more than 0.98. The ROC and the AUC reflect the good quality of QDA classifier. Robustness analysis of recognition is also conducted by adding some white noise artificially to the data and running the experiments in off-line mode. The recognition accuracy decreases when added noise into original data. When the signal noise ratio is no <100 : 0.75, the recognition accuracy is more than 96.0%, which is compared with real-time recognition with original data and without added noise. In conclusion, the recognition has robustness, when there is some noise (signal noise ratio ⩾ 100 : 0.75), according to this study. The contributions of the study are as follows. (1) In terms of a sensor, using just IMUs not multi-type sensors (for example sEMG and Mechanical sensor) fusion improves the integration and wearing convenience and maintain comparable recognition accuracy with multi-type sensors fusion at the same time. (2) On-board training solves algorithm integration problem with prosthetics. In addition, on-board training of model will lay a preliminary foundation for the model updating automatically which will make preparation for future recognition adaptation study.

### 4.4. Limitations

This study still need improvements in the further study. First, the on-board training time and recognition time are affected by the hardware performance and algorithm complexity. Higher performance processor, hardware acceleration, and new algorithms can be effective ways to decrease the training time and improve efficiency. Secondly, the recognition accuracy for locomotion modes still needs much improvement. This study focused more on the on-board training for real-time recognition, and we therefore have not conducted a transition study. Recognition delay for transition between various locomotion modes is an important parameter, and this was the subject of our previous study (Xu et al., 2018). In addition, this study is conducted in structured environment, which make good gait repeatability of prosthesis users. We need to explore whether and how the accuracy would be affected if the repeatability decreases. We should also combine the recognition with the prosthesis control to realize the better assistance for amputee in different locomotion modes in the future.

## 5. Conclusion

The paper puts forward an on-board training based on robotic transtibial prosthesis and develops the real-time human locomotion mode recognition based on the trained model. An interaction interface is designed for the study to collect sensor data, train models, and conduct recognition. The IMUs signals shows good gait repeatability. The on-board training time and recognition process time of three algorithms (SVM, QDA, and LDA) are used to evaluate the time performance. The real-time recognition accuracy based on QDA are 97.19 ± 0.36%. The study also achieves more than 95% recognition accuracy for each locomotion mode. The results show the on-board training is feasible and effective to recognize the amputee's locomotion mode with robotic transtibial prosthesis. Our study improves the integration and wearing convenience by just IMUs and maintains comparable recognition accuracy with multi-type sensors fusion at the same time and also solves algorithm integration problem with prosthetics.

## Data Availability Statement

The datasets generated for this study are available on request to the corresponding author.

## Ethics Statement

The studies involving human participants were reviewed and approved by the Local Ethics Committee of Peking University. The patients/participants provided their written informed consent to participate in this study.

## Author Contributions

DX and QW designed the research and wrote the paper. DX performed research and analyzed the data. All authors contributed to the article and approved the submitted version.

## Conflict of Interest

The authors declare that the research was conducted in the absence of any commercial or financial relationships that could be construed as a potential conflict of interest.
